# Editorial: Emerging Cellular Stress Sensors in Neurological Disorders: Closing in on the Nucleolus and the Primary Cilium

**DOI:** 10.3389/fncel.2020.00064

**Published:** 2020-03-20

**Authors:** Rosanna Parlato, Kerry L. Tucker

**Affiliations:** ^1^Institute of Applied Physiology, University of Ulm, Ulm, Germany; ^2^Institute of Anatomy and Cell Biology, Department of Medical Cell Biology, University of Heidelberg, Heidelberg, Germany; ^3^Department of Biomedical Sciences, Center for Excellence in the Neurosciences, College of Osteopathic Medicine, University of New England, Biddeford, ME, United States

**Keywords:** primary cilia, nucleolus, autophagy, cellular stress, cell homeostasis

The primary cilium and the nucleolus represent signaling hubs that regulate cell homeostasis and stress responses. Primary cilia have long been considered vestigial organelles but are now viewed as sensory antennae that transduce extracellular signals, including mechano- and chemo-transduction. The nucleolus is traditionally viewed as the site where ribosomal RNA synthesis and ribosome biogenesis occurs, which is now considered both a sensor and a mediator of the cellular stress response. Both organelles transduce developmental and homeostatic pathways, including Wnt signaling, mechanistic target of rapamycin (mTOR) and autophagy. Both organelles adjust their structure and function in response to changes not only in the extracellular environment but also in the intracellular milieu. Interestingly, neither are typical membrane-bound organelles, and they both adapt their structure in a dynamic fashion, suggesting a crucial role in cell homeostasis. These conceptual similarities, along with the newly discovered impact of deficits in primary ciliary and nucleolar function on neuronal homeostasis, raised the idea of the first collection of reviews and original articles addressing primary cilium- and nucleolus-dependent mechanisms in normal neuronal function and neurological disorders.

The Research Article by Monaco et al. describes a novel method to differentially isolate primary cilia from motile cilia by flow cytometry. Differently from motile cilia, primary cilia express type III adenylyl cyclase (AC3), a primary cilium-localized, cAMP-generating enzyme, and prominin, a glycoprotein typical of neural stem cells. Intriguingly, different populations of primary cilia characterized by the expression of specific receptors in an age-dependent fashion are identified here, validating this method for the investigation of primary cilia-dependent signaling pathways in health and disease conditions.

A second Research Article by Zhou et al. focuses on AC3 for its fascinating genetic association in human studies with autistic spectrum and major depressive disorders. The authors use mouse knockout models of the AC3 gene to look at differences in expression of phosphorylated protein isoforms in the brains of control and AC3-knockout mice. Interestingly, the authors also distributed their data sets by sex, and came up with hundreds of sex-specific phosphoprotein expression patterns, one third of which have shown association with autism.

The mini-review by Sarkisian and Semple-Rowland looks at connections between primary cilia and glioma. Gliomas are tumors arising from glial cell populations in the brain and spinal cord, comprising the majority of malignant brain tumors. The authors focus upon the most aggressive tumor type, the glioblastoma, and examine correlations and causative experiments that have been recorded and performed, respectively, to test whether primary cilia influence tumor development and progression. The data are decidedly mixed and do not seem to deliver a consistent answer whether tumor cells are ciliated, and conversely, the tumor identity of ciliated cells identified in biopsies. This uncertainty is mirrored by functional experiments, showing that either the presence or the absence of primary cilia on tumor precursor cells can promote tumor progression. In a second mini-review, the authors Park et al. make an excellent summary of the multiple, diverse, essential functions that primary cilia have in the formation of the brain, including patterning, layering, and neuronal migration within the forebrain, expansion of precursor pools in the cerebellum, and more subtle effects observed in learning and memory. They touch upon the best-investigated pathways, including Hedgehog, Wnt, mTOR, and they even discuss autophagy in this context.

In the Brief Research Report by Mustafa et al. we present the initial characterization of a mutant mouse lacking primary cilia in dopaminoceptive neurons. These models will be useful to investigate the impact of primary cilia in diseases affecting the dopamine system, such as Huntington's disease (HD). We show that either altered structure or loss of primary cilia is associated with increased mTOR activity in a progressive mouse model of HD. In addition loss of primary cilia results in bigger mutant Huntingtin nuclear inclusions, suggesting a more advanced pathological stage. Future studies should address the disease phenotype at later stages. A second Brief Research Report by Lucarelli et al. looks at the distribution of primary cilia in a murine model of the neurodegenerative Niemann-Pick C1 (NPC1) disease. NPC1 is characterized by biochemical changes in lipid and cholesterol metabolism resulting in dopamine imbalances and corresponding locomotor defects. In their mouse model, the authors observe a decrease in dopamine transporter expression, and also an increase in the number of primary cilia, in the dorsal striatum. In line with this concept, both reports strongly suggest that primary cilia are involved in homeostatic responses to changes in striatal dopamine. Future studies should address the impact of primary cilia of mTOR dysregulation and autophagy in neurodegeneration.

On the other side, the excellent Review by Pfister provides an outstanding overview of the regulation of autophagy by the second stress sensor organelle object of this Research Topic, the nucleolus. The author discusses recent findings on the crosstalk between mTOR signaling and nucleolar activity, and how autophagy represents a response to nucleolar stress in numerous human diseases. She provides an unprecedented outline of nucleolar proteins functioning in autophagy regulation. A second exciting Review by Latonen provides a comprehensive overview of the most recent functions discovered for the nucleolus in neuronal homeostasis. The author identifies the nucleolus as a central hub of cellular proteostasis and focuses on its important new role in cell homeostasis, affected by changing its material properties. The role of nucleolar aggresomes is discussed as a potential mechanism of nuclear aggregate accumulation common to many neurodegenerative diseases, such as HD and amyotrophic lateral sclerosis. This Review also presents the emerging role of long non-coding RNA in ribosomal RNA transcription and stress response.

Although there is no current evidence that primary cilia and nucleoli are interdependent, this Research Topic poses the provocative thought that the structure and function of these two key stress sensors and mediators might be connected. This collection promises to inspire original ideas and innovative studies, to provide a deeper understanding of physiological processes and disease mechanisms, and to identify new strategies for the maintenance of neuronal health and disease treatment.

## Author Contributions

RP and KT drafted the manuscript.

### Conflict of Interest

The authors declare that the research was conducted in the absence of any commercial or financial relationships that could be construed as a potential conflict of interest.

